# Higher rate of long-term serologic response of four double doses vs. standard doses of hepatitis B vaccination in HIV-infected adults: 4-year follow-up of a randomised controlled trial

**DOI:** 10.1186/s12981-019-0249-8

**Published:** 2019-11-11

**Authors:** Romanee Chaiwarith, Jutarat Praparattanapan, Wilai Kotarathititum, Jiraprapa Wipasa, Kanokporn Chaiklang, Khuanchai Supparatpinyo

**Affiliations:** 1 Division of Infectious Diseases, Department of Medicine, Faculty of Medicine, Chiang Mai University, Chiang Mai, Thailand; 2 Research Institutes for Health Sciences, Chiang Mai University, Chiang Mai, Thailand

**Keywords:** Hepatitis B vaccination, HIV infection, Immunogenicity, Four doses, Four double doses

## Abstract

**Background:**

We previously reported that four doses or four double doses of hepatitis B vaccination regimens could not significantly increase a response rate compared with standard doses. However, the antibody levels were higher in the four doses and four double doses groups. This study followed those patients for at least 3 years and aimed to evaluate the immunogenicity of the three vaccination regimens.

**Methods:**

HIV-infected adults who had CD4+ cell counts > 200 cells/mm^3^, undetectable plasma HIV-1 RNA, and negative for all hepatitis B virus markers were randomly assigned to receive one of three recombinant vaccines (Hepavax-Gene^®^ Berna, Korea) regimens: 20 μg IM at months 0, 1, and 6 (standard doses group, n = 44), 20 μg IM at months 0, 1, 2, 6 (four doses group, n = 44), or 40 μg IM at months 0, 1, 2, and 6 (four double doses group, n = 44) between February 2011 and May 4, 2012. Of 132 participants, 126 were evaluated from August 2015 to January 2016; 42 in the standard doses, 43 in the four doses, and 41 in the four double doses groups.

**Results:**

At a median duration of 49.7 months (range 46.7–53.7) after completion of the primary vaccination schedule, the percentages of responders with anti-HBs ≥ 10 mIU/mL were 57.1% (95% CI 41.5–72.8%) in the standard doses group; 76.7% (95% CI 63.6–89.9%) in the four doses group (*P *= 0.067 vs. the standard doses group); and 80.5% (95% CI 67.8–93.2%) in the four double doses group (*P *= 0.033 vs. the standard doses group). Factors associated with a responder were the vaccination schedule (either four doses or four double doses groups) and a younger age.

**Conclusions:**

Despite the highly effectiveness of the standard hepatitis B vaccination regimen at 6 months after completion, the long-term immunogenicity was lower than the four double doses regimen among HIV-infected adults with CD4+ cell counts > 200 cells/mm^3^ and undetectable plasma HIV-1 RNA. The standard vaccination regimen may not be the best strategy to provide long-term immune response against hepatitis B virus among HIV-infected individuals.

*Trial registration* NCT1289106, NCT02713620

## Background

HIV-infected patients have been noted to have a poorer response to the hepatitis B virus (HBV) vaccination than HIV-uninfected individuals in terms of antibody levels and duration of serologic responders [1–3]. The response rate to vaccination ranged between 38 and 95% depending upon the vaccination schedule, CD4 cell counts, achievable of undetectable HIV-RNA, HCV co-infection, or occult HBV infection [4–14]. We conducted a randomised controlled trial among HIV-infected patients aged ≥ 18 years old, with a CD4+ cell count > 200 cells/mm^3^, undetectable plasma HIV-1 RNA, negative for hepatitis B surface antigen (HBsAg), antibody to hepatitis B surface antigen (anti-HBs), and antibody to hepatitis B core antigen (anti-HBc), negative for antibody to HCV (anti-HCV) and had no active opportunistic infections at the time of screening [4]. The response rate to the standard hepatitis B vaccination regimen was 88.6% at 1 month after vaccine completion. This response rate is almost as high as that achieved in non-HIV healthy adults [3, 15]. The response rate was non-significantly lower than the four doses group and the four double doses group. However, the percentage of high-titer responders (anti-HBs ≥ 100 mIU/mL) was significantly higher among the four double doses group compared with the standard doses regimen [2, 4].

We, therefore, followed those patients for at least 3 years with the aim to evaluate the efficacy of HBV vaccination schedules using either four doses or four double doses compared with the current standard doses regimen in HIV-infected adults in Northern Thailand.

## Methods

We followed participants who participated in a randomised, open-label, controlled trial between February 4, 2011 and May 4, 2012 at Chiang Mai University Hospital, Chiang Mai, Thailand. In brief, HIV-infected adults aged ≥ 18 years old, who had a CD4+ cell count > 200 cells/mm^3^, undetectable plasma HIV-1 RNA, negative for HBsAg, anti-HBs antibody, and anti-HBc antibody, had no history of previous vaccine, negative for anti-HCV, and had no active opportunistic infections (at the time of screening) were randomised in a 1:1:1 allocation ratio by a block of six: 1) the standard doses group receiving three intramuscular injections of 20 μg of recombinant HBV vaccine (Hepavax-Gene^®^ Berna, Korea) at months 0, 1, and 6; or 2) the four doses group receiving four intramuscular doses of 20 μg of the same vaccine at months 0, 1, 2, and 6; or 3) the four double doses group receiving four intramuscular double doses (40 μg) at months 0, 1, 2, and 6 [4].

This study followed those participants for at least 3 years after the completion of the vaccine schedule. HBs antigen, anti-HBs antibody, anti-HBc antibody, and anti-HCV antibody were re-tested. Participants were excluded if they had a CD4+ cell count < 200 cells/mm^3^, or detectable plasma HIV-1 RNA, or positive for any of HBs antigens, anti-HBc antibody, and anti-HCV, or received an additional hepatitis B vaccine after the completion of those vaccination schedule. Written informed consent was obtained.

Hepatitis profile including HBsAg, anti-HBs antibody, anti-HBc antibody and anti-HCV were performed on collected sera at the Central Diagnostic Laboratory, Maharaj Nakorn Chiang Mai Hospital, using a standardized enzyme immunoassay (ARCHITECT System, Abbott, USA). The ARCHITECT HBsAg assay is a chemiluminescent microparticle immunoassay (CMIA) which uses microparticles coated with monoclonal anti-HBs for detection of HBsAg (specificity 99.87%; sensitivity 99.52%). The ARCHITECT Anti-HBs assay is a CMIA technology for the quantitative determination of Anti-HBs (specificity 99.67%; sensitivity 99.54%). The ARCHITECT Anti-HBc II and Anti-HCV assay is a CMIA for qualitative detection of anti-HBc (specificity 99.50%; sensitivity 100%) and anti-HCV (specificity 99.60%; sensitivity 99.10%), respectively. Samples were tested by technical staff blinded to vaccine group allocation.

The primary study was approved by the Research Ethics Committee 1 and this study was approved by the Research Ethics Committee 4, Faculty of Medicine, Chiang Mai University. The primary study was registered on ClinicalTrials.gov; NCT1289106 on February 1, 2011 and this study was retrospectively registered on ClinicalTrials.gov; NCT02713620 on March 21, 2016. All participants were recruited after ethical approval.

## Statistical analysis

The primary analysis required 41 participants in each group without lost to follow up to detect the differences between groups in terms of the percentages of responders (anti-HBs titers ≥ 10 mIU/mL) at 1 month after completion of vaccination schedule. This study followed those participants to determine the percentages of responders (anti-HBs titers ≥ 10 mIU/mL), high-titer responders (anti-HBs titers ≥ 100 mIU/mL), and anti-HBs titers at ≥ 3 years elapse since the last dose of vaccination schedule. Proportions of participants with responders, high-level responders between groups (i.e. the four double doses group vs. the standard doses group and the four doses group vs. the standard doses group) were compared using Chi square test or Fisher’s exact test for categorical data and Student’s t-test or Mann–Whitney *U* test for continuous data. Factors associated with seroprotection and achieving high-titer antibody were tested in univariate models. Factors with the P-value < 0.10 from univariate analysis were then tested in a multivariate logistic regression model using forward stepwise procedure. All statistical analyses were performed using Stata statistical software version 10.0 (Stata Statistical Software: Release 10.0, Stata Corporation, College Station, TX, 2007). A two-sided test was used to indicate statistical significance at a P-value of < 0.05.

## Results

Between August 2015–January 2016, 126 participants were enrolled; 42 in the standard doses group, 43 in the four doses group, and 41 in the four double doses group (Additional file 1: Fig. S1). The remaining six participants were referred to local hospitals and declined to participate in the study. The median duration from the completion of the vaccine schedule was 49.7 months (range 46.7, 53.7).

As previously reported, demographic and clinical characteristics of participants by vaccination regimen at the time of vaccination were similar in terms of ages, body mass index, creatinine clearance, nadir CD4 cell count, time elapsed since HIV diagnosis, antiretroviral regimens, history of drug resistance, HIV risk exposure, alcohol uses and underlying diseases including hypertension, and dyslipidemia. There were more males and a longer duration of combination antiretroviral therapy in patients in the four double doses group than the standard doses group. Patients in the standard doses group had a lower median CD4 cell count than the other two groups; [400 cells/mm^3^ (IQR 314, 558) in the standard doses group vs. 544 cells/mm^3^ (IQR 416, 731) in the four doses group vs. 544 cells/mm^3^ (IQR 410, 642) in the four double doses group]. The duration of suppressed plasma HIV-1 RNA was shorter in patients in the standard doses group than the other two groups [4].

The median CD4 cell count during this follow up period was 534 (IQR 449, 706) cells/mm^3^ in the standard doses group, 694 (IQR 553, 910) cells/mm^3^ in the four doses group (*P *= 0.003 vs. the standard doses group) and 625 (493, 774) cells/mm^3^ in the four double doses group (*P *= 0.237 vs. the standard doses group). All patients had undetectable HIV-1 RNA.

## Immunogenicity

**Fig. 1:**
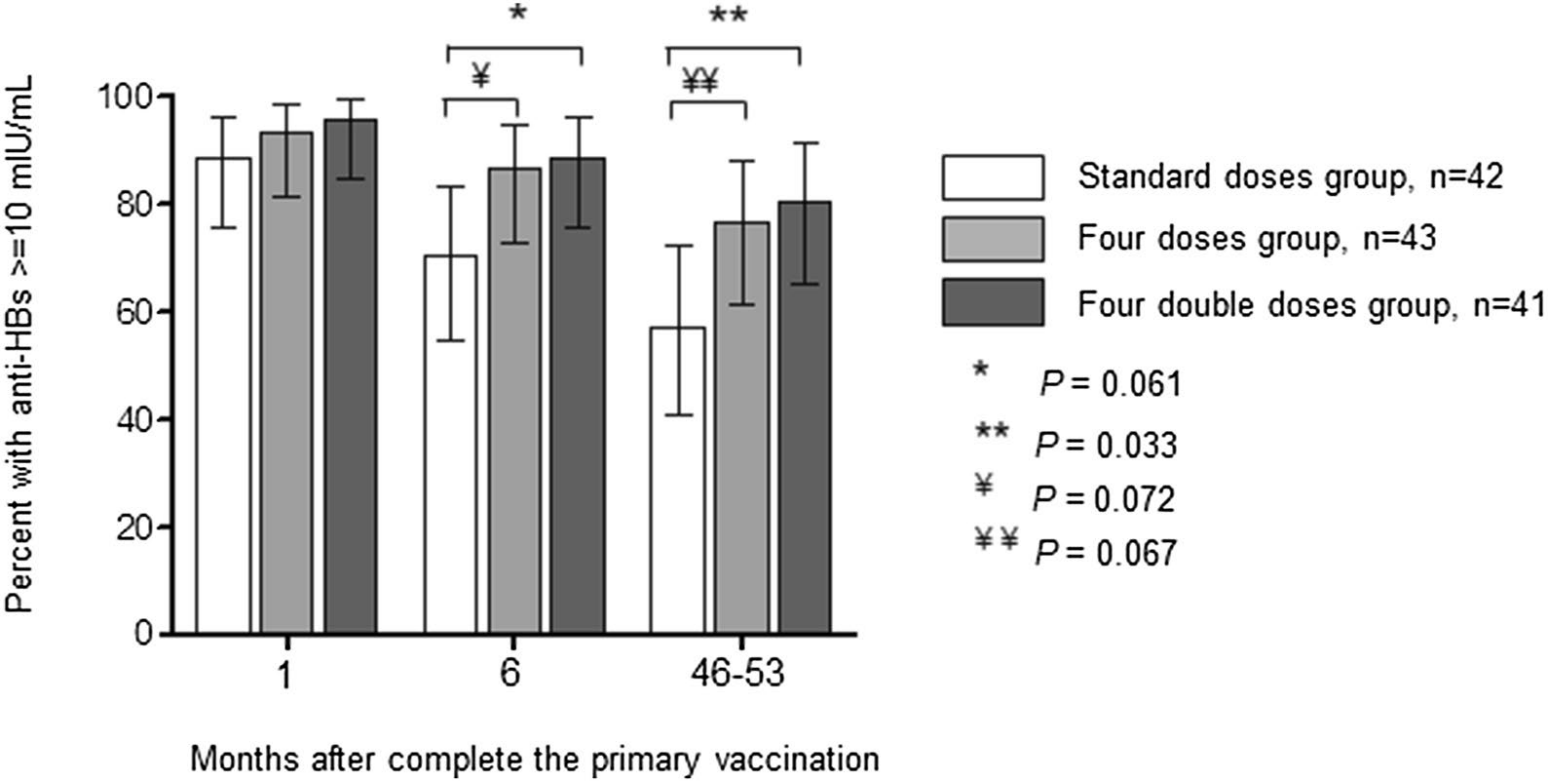
Percentages of responders (anti-HBs ≥ 10 mIU/ml) to hepatitis B vaccine by vaccination regimen

The percentages of responders (participants with anti-HBs titers ≥ 10 mIU/mL) were 57.1% (95% CI 41.5–72.8%) in the standard doses group; 76.7% (95% CI 63.6–89.9%) in the four doses group (*P *= 0.067 vs. the standard doses group); and 80.5% (95% CI 67.8–93.2%) in the four double doses group (*P *= 0.033 vs. the standard doses group), respectively (Fig. 1).

**Fig. 2:**
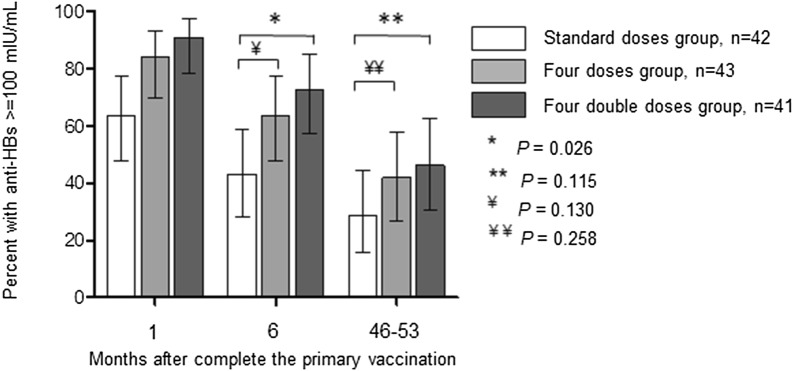
Percentages of high responders (anti-HBs ≥ 100 mIU/ml) to hepatitis B vaccine by vaccination regimen

The percentages of high-titer responders (anti-HBs ≥ 100 mIU/mL) were 28.6% (95% CI 14.3–42.8%) in the standard doses group; 41.9% (95% CI 26.5–57.2%) in the four doses group (*P *= 0.258) and 46.3% (95% CI 30.4–62.3%) in the four double doses group (*P* = 0.115 vs. the standard doses group) (Fig. 2).

**Fig. 3:**
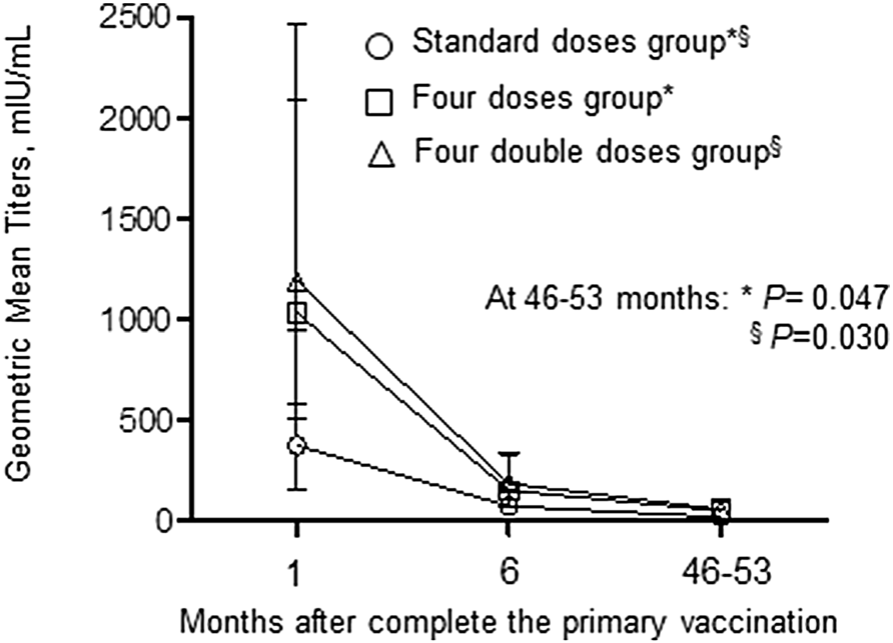
Geometric Mean Titers of anti-HBs antibody by vaccination regimen

The geometric means of anti-HBs titer were 19.8 mIU/mL (95% CI 9.8–39.8) in the standard doses group; 51.6 mIU/mL (95% CI 29.3–91.0) in the four doses group (*P *= 0.047 vs. the standard doses group); and 57.5 mIU/mL (95% CI 29.7–111.5) in the four double doses group (*P *= 0.030 vs. the standard doses group), respectively (Fig. 3).

## Factors associated with responders

Characteristics between responders and non-responders are shown in Table 1. Multivariate analysis revealed that factors associated with achieving a protective antibody level (anti-HBs ≥ 10 mIU/mL) were the vaccination schedule (OR for the four doses group vs. the standard doses group = 2.95, 95% CI 1.11–7.85, *P *= 0.030, OR for the four double doses group vs. the standard doses group = 3.35, 95% CI 1.22–9.19, *P *= 0.019), and younger age at the time of vaccination (the odds of achieving protective antibody level increase 39.5% for every 5 years younger, 95% CI 1.8–91.2%). No factors associated with achieving a high titer level (anti-HBs ≥ 100 mIU/mL) were demonstrated.

**Table 1:** Comparison of characteristics between responders and non-responders (anti-HBs ≥ 10 mIU/ml)

Predictive factors	Responders (n = 90)	Non-responders (n = 36)	P-value
Age at primary vaccination, years (mean ± SD)	40.9 ± 6.1	43.3 ± 7.4	0.066
Male	23 (25.6%)	9 (25.0%)	0.948
Underlying diseases			
Diabetes	10 (11.1%)	6 (16.7%)	0.397
Dyslipidemia	12 (13.3%)	8 (22.2%)	0.217
Hypertension	6 (16.7%)	15 (16.7%)	1.000
Risk of HIV acquisition			0.443
Heterosexual	34 (94.4%)	87 (96.7%)	
Homosexual (MSM)	1 (2.8%)	1 (1.1%)	
IVDU	0	1 (1.1%)	
Blood transfusion	1 (2.8%)	0	
Unknown	0	1 (1.1%)	
cART regimen			0.664
NNRTI-based	81 (90%)	33 (91.7%)	
PI-based	8 (8.9%)	2 (5.6%)	
Others	1 (1.1%)	1 (2.8%)	
History of HIV-drug resistance	6 (6.7%)	3 (8.3%)	0.743
Duration of cART, years (median, IQR)	7.0 (2.7, 8.6)	6.8 (5.2, 8.5)	0.797
Initial CD4+ cell count, cells/mm^3^ (median (IQR))	482 (395, 627)	407 (340, 631)	0.315
Current CD4+ cell count, cells/mm^3^ [median (IQR)]	609 (480, 818)	588 (493, 744)	0.574
Vaccination schedule			0.040
Standard doses	24 (26.7%)	18 (50.0%)	
Four doses	33 (36.7%)	10 (27.8%)	
Four double doses	33 (36.7%)	8 (22.2%)	

*cART* combination antiretroviral therapy, *IQR* interquartile range, *IVDU* intravenous drug use, *mm*^*3*^ cubic millimeter, *MSM* men who have sex with men, *NNRTI* non-nucleoside reverse transcriptase inhibitor, *PI* protease inhibitor, *SD* standard deviation

## Discussion

Among HIV-infected individuals with CD4 cell count ≥ 200 cells/mm^3^ and undetectable HIV-1 RNA, the response rate to the standard HBV vaccination schedule ranged between 40 and 71% [8, 9, 13, 16]. A number of studies try to find the best strategy to improve seroprotection against HBV among HIV-infected individuals. Those strategies included increasing the dose, the frequency, both the dose and frequency of the vaccination schedule, route of vaccine administration, e.g. intradermal route, or adding GM-CSF to the vaccine regimen [6, 8, 9, 11, 12, 17, 18]. A randomised controlled trial conducted by Launay et al. demonstrated that a 4-double-dose schedule produces higher anti-HBs titers, seroconversion rate, and high responder rate than the three standard doses [9]. Another study conducted by Fosceca et al. demonstrated that three double doses non-significantly improved the seroconversion rate [8]. The primary analysis of this study in which the primary endpoint was the percentage of responders at 1 month after the last dose of vaccination (month 7) also demonstrated the same findings as Launay et al. [4]. Up to the present, no randomised controlled trial demonstrated the superiority of four-double doses over four-standard doses or three-double doses.

This study followed the participants in the primary analysis with the median follow-up time of 49.6 months addressed the importance of high anti-HBs titers after primary vaccination. Studies of long-term seroprotection rates have been reported [19–23]. The longer time elapsed since completion of the vaccination schedule, the lower the rate of seroprotection existed. Factors associated with the persistence of anti-HBs were higher CD4 cell counts at the time of vaccination and anti-HBs levels after vaccination [19–23]. The median time to loss of seroprotection was 2.0, 3.7, and 4.4 years for those with anti-HBs titers of 10–100 IU/L, > 100–1000 IU/L, and > 1000 IU/L at primary vaccination, respectively [21]. Another study among 119 patients with the median CD4 counts of 506 cells/mm^3^, 70% and 27% of participants retained seroprotection at 36 and 84 months after vaccination, respectively [22]. The latter study suggested re-checking HBs antibody titer 5 years after completing the vaccination schedule. A secondary analysis of a randomised controlled study conducted in France by Launay et al. found that at month 42, the percentage of responders was 41% (95% CI 33–49%), and 71% (95% CI 64–79%) among those who received three standard doses and four double doses, respectively. Fifteen percent of patients lost their protective antibody level at 8.7 and 33.1 months in three-standard doses and four double doses, respectively [20]. Our study confirmed the findings from previous reports. With the median time of 49.6 months, the seroprotection rates were 57.1% (95% CI 41.5–72.8%) and 80.5% (95% CI 67.8–93.2%) among those who received standard doses and four double doses, respectively. Factors associated with seroprotection were younger age at vaccination and the vaccination schedule either four standard doses or four double doses. In the primary analysis, the GMT at 1 month after the vaccine series was non-significantly higher in four standard doses vs. standard doses, but significantly higher in four double doses vs. standard doses. This corresponded to the seroprotection rate of the three regimens. The follow-up period of the primary study confirmed that the percentage of responders was higher in the four double doses group compared with the standard doses group. Although the response rate was higher in the four doses group (76.7%) compared with the standard doses group (57.1%), the magnitude did not reach statistical significance. The same trend was shown in the percentage of high-titer response rate. As this study was not designed to compare the four double doses and the four doses groups, we cannot draw any conclusions between these two groups.

Based on the results of this study along with other previous reports, the four double doses vaccine schedule increased long-term protective antibody levels as compared with the standard doses schedule. However, a randomised controlled trial is still needed to determine whether the four double doses schedule is superior to the four doses schedule or three-double dose schedule as the higher antibody titer after primary vaccination conveyed longer seroprotection.

Our study had some limitations. First, the study included only HIV-infected adults with CD4+ cell counts > 200 cells/mm^3^ and undetectable plasma HIV-1 RNA, so the results could not be generalized to other groups of HIV-infected individuals. Second, we did not design the trial to compare the response rates between the four double doses group and the four doses group, the sample size was inadequate to detect the difference if it existed. Third, as we performed the antibody titer at a specific time point, we were unable to calculate the median time to loss of seroprotection. The appropriate time point to re-assess anti-HBs titers could not be addressed from this study.

In conclusion, anti-HBs titers were significantly higher with four-double doses schedule than the standard doses and therefore, conveyed a longer-term protection.

## Supplementary information

**Additional file 1: Fig. S1.** Consort diagram of participants.

## Data Availability

Data will not be shared as the local IRB has no policy to share the data without prior permission.

## Abbreviations

anti-HBc: anti-hepatitis B core
anti-HBs: anti-hepatitis B surface
CMIA: chemiluminescent magnetic microparticle immunoassay
GMT: geometric mean titer
HBs: hepatitis B surface
HBV: hepatitis B virus
HCV: hepatitis C virus
HIV: human immunodeficiency virus
IM: intramuscular
IQR: interquartile range
mIU/mL: milli-international units per milliliter
mm^3^: cubic millimeter
OR: odds ratio
RNA: ribonucleic acid
SD: standard deviation
