# Supplementary figures and images for: Higher rate of long-term serologic response of four double doses vs. standard doses of hepatitis B vaccination in HIV-infected adults: 4-year follow-up of a randomised controlled trial
Source: AIDS Res Ther. 2019 Nov 11;16:33. doi: 10.1186/s12981-019-0249-8 (PMC6844022; doi:10.1186/s12981-019-0249-8)

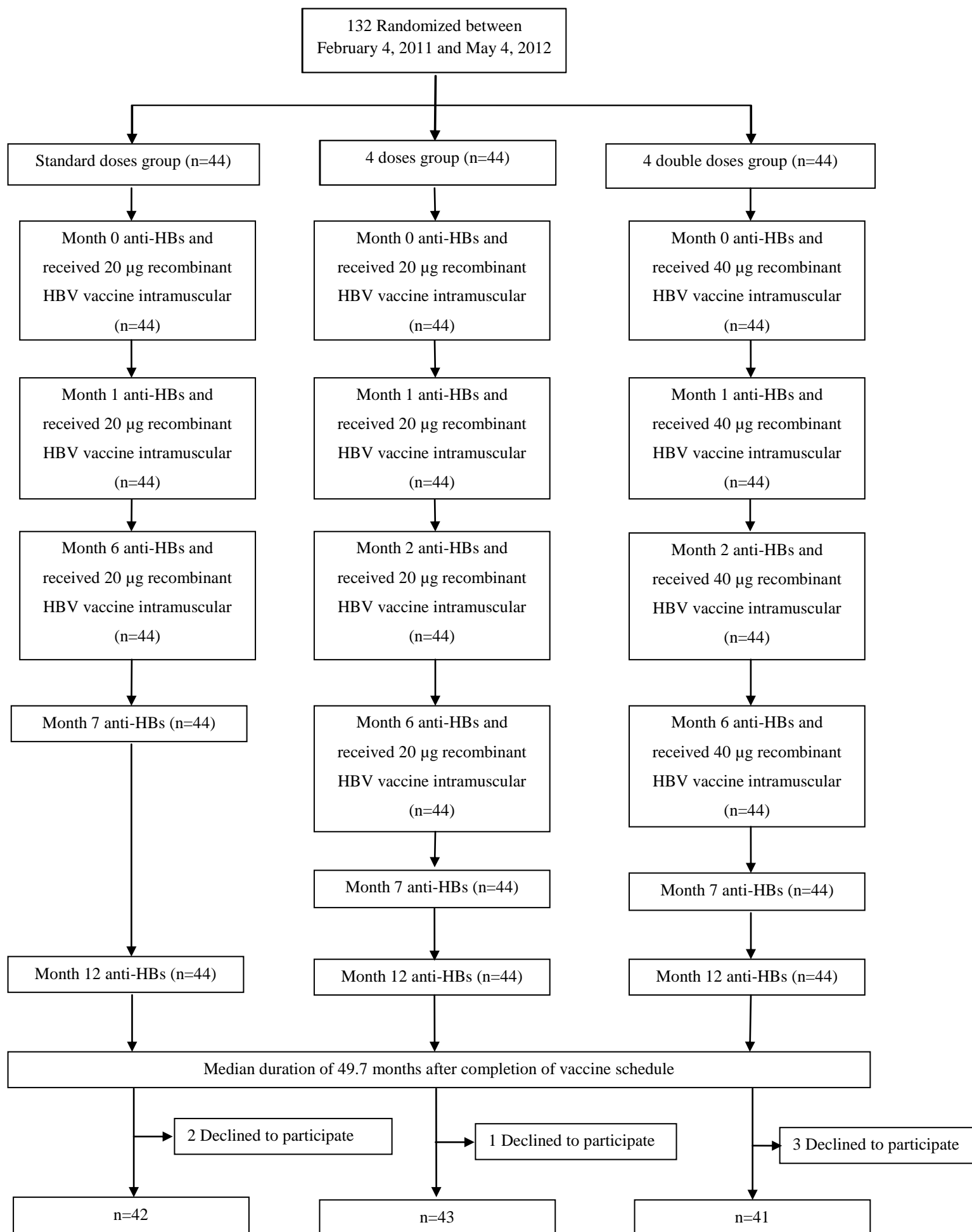

Fig. S1. Consort diagram of the study participants

Supplement: Supplementary file 1 — Additional file 1: Fig. S1. Consort diagram of participants. [file 12981_2019_249_MOESM1_ESM.pdf]
